# Popular music and movies as autobiographical memory cues

**DOI:** 10.3758/s13421-025-01765-2

**Published:** 2025-08-08

**Authors:** Julien Hanson, Jessica Frame, Elena Bai, Kendra Mehl, Kelly Jakubowski, Amy M. Belfi

**Affiliations:** 1 Department of Psychological Science, Missouri University of Science and Technology, 135 H-SS, 500 W. 14th St, Rolla, MO 65409 USA; 2 Department of Music, Durham University, Durham, UK

**Keywords:** Autobiographical memory, Music, Movies, Emotion, Reminiscence bump

## Abstract

Research on music-evoked autobiographical memories has grown rapidly in recent years, suggesting that music can be an effective stimulus for cueing memories from one’s life. One challenging aspect of this type of research is creating a stimulus set that is effective at cueing autobiographical memories in a wide range of individuals. The present work sought to address this issue by creating a normed stimulus set of popular music and popular movie cues. In addition to this methodological aim, we had an empirical aim to identify differences between autobiographical memories cued by music and movies. Participants (*N* = 248) either listened to excerpts of popular music or viewed clips of popular movies. After each stimulus, participants rated it on several dimensions, including emotional valence, emotional arousal, familiarity, and autobiographical salience. Results indicated that certain songs and movies are autobiographically salient across a wide age range of participants. Additionally, we identified that musical cues show a significantly more pronounced reminiscence bump than movie cues, suggesting that music from the reminiscence bump period of life is more effective at triggering memories. Overall, these data provide an important resource for researchers wishing to use popular media to cue autobiographical memories, as well as indicating differences between memories cued by music and movies.

**Supplementary Information:**

The online version contains supplementary material available at 10.3758/s13421-025-01765-2.

Autobiographical memories are personal memories of events that occurred during one’s life (Conway & Pleydell-Pearce, 2000). Autobiographical memories can arise spontaneously, sometimes without a clear cue. Other times, they can be cued by a specific stimulus, such as a sound, smell, sight, or even an internal thought or feeling (Berntsen, 2021). In recent years, there has been a growing interest in studying music as a cue for autobiographical memories (Belfi & Jakubowski, 2021). Research has indicated that music can effectively cue autobiographical memories and that music-evoked autobiographical memories (MEAMs) tend to be highly emotional (Janata et al., 2007). More recent work suggests that MEAMs happen quite frequently during everyday life, with individuals reporting near-daily occurrences (Jakubowski & Ghosh, 2019). Additionally, the growing interest in music as an autobiographical memory cue is likely driven by increasing evidence suggesting that music is a particularly effective memory cue for individuals with Alzheimer’s disease (Baird & Samson, 2009; El Haj et al., 2012, 2013).

While initial work on music and autobiographical memory indicated that music *can* cue autobiographical memories, more recently, researchers have started comparing music to other memory cues to identify whether memories evoked by music differ in some way. For example, our prior work investigated music compared to pictures of famous persons: Although musical cues evoked fewer autobiographical memories than pictures of famous people, these memories contained more internal and perceptual details, indicating a higher degree of episodic richness (Belfi et al., 2016, 2020). Subsequent research found that MEAMs were more episodically rich than face-evoked memories, even after controlling for the level of effort required to retrieve those memories (Belfi et al., 2022). That is, whether the stimulus evoked a memory spontaneously or effortfully, musical cues still elicited memories that contained a higher level of episodic richness than face cues.

Other studies have tested a wide range of sensory cues in comparison to music. One study looked at the specificity and frequency of memories evoked by music and photographs of famous events among healthy aging adults and adults with Alzheimer’s disease (Baird et al., 2018). Results indicated that popular music was more likely to evoke memories in individuals with Alzheimer’s disease than photos of famous events. Other relevant work compared retrieval characteristics, content, and emotions of music- and television-evoked autobiographical memories (Jakubowski et al., 2021). Memories evoked by musical cues were seen as more vivid, emotionally intense, and of more personal significance than memories evoked by television shows. However, it is important to note that this study did not actually present participants with stimuli; instead, it was a survey in which participants reported prior experiences of music- and television-evoked memories.

In addition to visual and audiovisual cues, other work has compared music to word cues, finding that MEAMs contained more auditory and motor-perceptual details (Zator & Katz, 2017). Music has also been compared to food: In a recent study, participants recorded occurrences of music- and food-evoked memories in a diary over a period of several days (Jakubowski et al., 2023). Music triggered memories more frequently than food, and these memories were rated more personally significant. Overall, a growing body of work has compared music to other sensory cues (e.g., images, television, food). This work has suggested that MEAMs differ from memories evoked by other sensory cues in a number of ways, including the frequency of memories evoked, the content of those memories, and the phenomenological characteristics of recalling those memories. There are multiple potential theoretical explanations for why music might be an effective memory cue. For one, music may serve as an effective contextual cue, given its ubiquitous nature and its frequent presence in the “background” of many life events. Secondly, the emotional potency of music has been posited as one reason why it might be a highly effective memory cue, given the fact that emotion can often facilitate memory retrieval.

In addition to these characteristics, other work has investigated the shape of the reminiscence bump for music-evoked memories. The “reminiscence bump” describes the phenomenon whereby, when participants are asked to recall memories from their lifetime, the frequency of memories tends to be highest during adolescence and early adulthood. When looking at the frequency distribution of reported memories, therefore, the shape forms a “bump,” with the highest frequency of memories occurring during this period of life (Janssen et al., 2011; Rubin & Schulkind, 1997). This bump tends to emerge when looking at the data in aggregate, across many participants, rather than at the individual level. The reminiscence bump finding has traditionally been in response to verbally prompted memories, but more recently, researchers have investigated reminiscence bumps for MEAMs.

Early work on this topic suggested that music displays both a standard reminiscence bump (i.e., during adolescence and early adulthood) as well as a “cascading reminiscence bump” (Krumhansl & Zupnick, 2013). In initial work on this topic, younger adults listened to clips of popular music from the 1950s to the present and reported any autobiographical memories that were evoked. Results indicated a traditional reminiscence bump (i.e., a peak for participants’ adolescence and early adulthood) but also a secondary bump around when the participants’ parents were adolescents. Other work has compared the shape of reminiscence bumps for music versus other cues: In one task, participants viewed the titles of popular songs and were asked to indicate the degree to which the songs evoked autobiographical memories (Jakubowski et al., 2020). The authors found that the music-evoked reminiscence bump peaked at a similar age to word-cued memories (around age 14). A similar study compared music to films –the researchers presented participants with a list of titles of songs and movies and asked participants to pick which were most personally significant (Rathbone et al., 2016). They found a more pronounced reminiscence bump for music than for films.

It is worth noting, however, that the way “reminiscence bump” has been defined in music research is somewhat different from the definition in other memory research more broadly. Traditionally, the reminiscence bump has been defined based on the age of the *memories*—that is, the memories are of events that took place during adolescence and early adulthood. In music-evoked autobiographical memory research, however, the term “reminiscence bump” has tended to refer to the age of the *stimulus*—that is, memories evoked by songs that were popular during adolescence and early adulthood. It is not only music research that defines the reminiscence bump in this way but also research on memory for cultural products more broadly: For example, prior work has investigated this type of reminiscence bump for films (Holbrook & Schindler, 1996; Rathbone et al., 2016), celebrities (Holbrook & Schindler, 1994), and even football players (Janssen et al., 2012). Here, we define reminiscence bump in this second manner, in line with prior research on music and cultural products. However, we do not assume that just because a stimulus comes from a certain lifetime period, that the memory also comes from that period. For example, in the case of cascading reminiscence bumps, it is possible to have memories evoked by songs that were released prior to one’s birth.

It is also important to note the variety of methods used in prior studies of MEAMs: Some studies have collected data during everyday life, others present lists of titles, and others have presented the actual sensory stimuli themselves (i.e., musical clips and other comparison stimuli). For studies that did present musical stimuli, these stimulus sets have been created in an ad hoc manner: Some studies presented composite stimuli, with several songs merged together (Krumhansl & Zupnick, 2013) whereas others have presented individual song clips (Belfi et al., 2016). Studies that wish to investigate a large range in participant ages or the shape of a reminiscence bump would require gathering many thousands of musical clips from across the past decades. This is quite a demanding task for researchers, so there is great need for a normed stimulus set of musical clips that researchers can use in this area. To this end, one of the goals of this paper is a methodological one, which is to provide a stimulus set with normative ratings for the purpose of evoking autobiographical memories.

In the present work, we therefore sought to collect a large sample of responses for musical cues and one comparison cue. In this case, we chose popular movies. Popular movies have only been compared to music in prior work via titles rather than the actual stimuli themselves (Rathbone et al., 2016). However, they are an apt comparison cue in that both music and movies are temporally evolving (i.e., not static such as images or words), ubiquitous parts of popular culture, and can be easily tied to a specific time period. There are currently some existing stimulus sets of video clips; however, they are either unfamiliar news clips (Samide et al., 2019) or are movie clips that were selected to evoke specific emotions rather than memories (Schaefer et al., 2010; this stimulus set was also normed on a French sample of participants). There are no currently existing normed large stimulus sets of popular music and movie cues for the purpose of evoking autobiographical memories for a US-based audience. Jakubowski and colleagues  (2020) provide normative ratings on a set of song *titles*; however, their data are also normed for a French sample rather than a US-based one.

Therefore, one of the goals of the present study is to provide a resource for future researchers who wish to use musical and/or film stimuli to trigger autobiographical memories. Given the dramatic increase in interest in this topic and the challenge for each individual research team to compose a stimulus set of music that could be effective for evoking memories in a large range of people, we sought to provide this resource for the field. This will also allow for comparability and replicability across different studies. We hope that these stimulus sets reduce the burden of conducting this type of research. In addition to this methodological goal, we also have an empirical goal—namely, the present study seeks to add to the growing body of literature comparing MEAMs to memories evoked by other cues. While previous research has compared music to films (Rathbone et al., 2016) and television (Jakubowski et al., 2021), both these studies used a survey method in which participants were not presented with the sensory stimuli themselves. Here, we sought to investigate whether the memory characteristics, phenomenological experience, and reminiscence bump shapes differed between autobiographical memories evoked by music and movie cues.

## Study 1: Characterizing popular music cues

In Study 1, we sought to characterize a set of musical stimuli for use in future autobiographical memory research. These stimuli have been used in a series of prior studies (Belfi et al., 2016, 2022), and here we sought to provide normative ratings for these stimuli for other researchers to use in future work.

### Method

#### Participants

Participants (*N* = 120; 59 women, 60 men, one nonbinary) were recruited online using Prolific, an online platform for participant recruitment (Palan & Schitter, 2018). Participation was restricted to workers who are native English speakers, live in the United States, and have completed at least 100 previous Prolific tasks and obtained approval ratings of at least 95%. Our goal was to recruit participants with evenly distributed ages between 20 and 85 years of age using a stratified sampling method. Approximately 20 participants were recruited in each of the following 10-year age bins: 20–29, 30–39, 40–49, 50–59, 60–69, and 70–85 years old. Sample size was based on our prior work collecting normative data for musical stimulus sets (Belfi & Kacirek, 2021).

Additionally, we sought to have an adequately powered sample size to test for differences in subjective ratings and memory qualities between groups (music and movies). To this end, we conducted an a priori power analysis using G*Power software (Faul et al., 2007). We identified our estimated effect size from our prior work comparing the vividness of memories evoked by music to those evoked by images (which reported a Cohen’s *d* = 0.55). Assuming this effect size, we conducted an a priori power analysis to determine the sample size necessary to detect a significant main effect of stimulus group (music vs. movies). This indicated that a sample size of 73 participants per group would be adequate to detect an effect of this size, so we therefore feel that our sample is adequately powered to test between movies and music groups (see Study 3 for these comparisons between stimulus groups). To summarize, our sample size was determined to both allow for the adequate sampling across age groups for the purposes of normative data collection, as well as for adequate statistical power to test for effects between music and movie stimulus groups.

#### Stimuli

Stimuli consisted of popular musical cues used in our previous research on music-evoked autobiographical memories (Belfi et al., 2016, 2022). Briefly, songs were chosen from the Billboard Hot 100 year-end charts from the years 1950-2020. To minimize participant fatigue, we chose the top *two* songs for each year to include in our stimulus set that would be presented to participants. Therefore, the total set consisted of 144 stimuli (two songs per year for 72 years). Due to a programming error, three songs were not presented to participants, leaving 141 total stimuli with rating data. Each song was clipped to a 15-second long segment that corresponded to the chorus or other highly recognizable part of the song. Again, to minimize participant fatigue, stimuli were assigned to one of four lists. Each list contained one clip from every other year, for a total of 36 songs per list. For the full list of all stimuli, including which list each clip was included in, please see the Supplemental Materials.

#### Procedure

All procedures were conducted in compliance with the American Psychological Association Ethical Principles and were approved by the Institutional Review Board at the University of Missouri. The task was presented using Gorilla, an online platform for designing psychological experiments. First, participants read a general description of the study and provided their consent to participate. Then, they were randomly assigned to receive one of the four sets of stimuli; in total, each participant completed 36 trials. Each trial started with a fixation cross for 500 ms. Following this, the song clip was played. The clips were played in a random order. Clips automatically started playing following the fixation and were only played once.

After each clip, participants completed a series of questions: First, they were asked to provide the *name* of the song by typing the name into a text box (“What is the name of this song?”). Next, they rated their *familiarity* with the song (“Rate your familiarity with this song”) on a 6-point scale ranging from certain familiarity (a 6 on the scale) to certain unfamiliarity (a 1 on the scale). On this scale, a rating of 3 or below indicated that the participant was not familiar with the song clip, whereas a rating of 4 or above indicated that the participant had some degree of familiarity with the melody. A rating of 1 indicated “unfamiliar, completely certain” while a rating of 3 indicated “unfamiliar, somewhat certain.” A rating of 6 indicated “familiar, completely certain” while a rating of 4 indicated “familiar, somewhat certain.” This type of rating scale has been used in our prior work on familiarity of musical melodies (Belfi & Tranel, 2014).

Next, participants rated the emotional *valence* of the song (“How positive or negative is the emotion expressed by this musical clip?”; 1–7-point scale; anchors were “very negative” and “very positive”), the emotional *arousal* of the song (“How relaxing or stimulating is this musical clip?”; 1–7-point scale; anchors were “very relaxing” and “very stimulating”), and how much they *liked* the song (“How much did you like this musical clip?”; 1–7-point scale; anchors were “dislike very much” and “like very much”). Next, participants indicated whether the song evoked *autobiographical memories* (“Did this music clip evoke personal memories of events from your life?”; Yes/No response). If participants said “no” to this question, they proceeded to the next trial. If they said “yes,” they then rated the *strength* and *vividness* of the memories evoked (“How strong and vivid were the memories that were evoked by this clip?”; 1–7-point scale; anchors were “very weak/vague memories” and “very strong/vivid memories”). They then provided a brief written description of the memory in a text box (“Please briefly describe the memory or memories that were evoked by the clip”). Following all 36 trials, participants completed a brief demographics questionnaire, which asked their gender, date of birth, as well as the dates of birth for up to two parents or guardians.

#### Data quantification

For naming responses, one experimenter read through all typed responses and manually scored them as correct or incorrect, as in prior research (Belfi & Tranel, 2014). If a participant’s response matched the correct title of the song, it was scored as a 1 for correct; if it did not match, it was scored a 0 for incorrect. For any ambiguous or unclear cases, a second rater was asked to rate the item. If the two raters matched, that was the given score. If the two raters diverged, they discussed and came to a consensus. For the autobiographical memory Yes/No question, all “yes” responses were converted to 1 and “no” responses were converted to 0.

#### Analysis

One of the goals of the present project was to assemble a normed set of popular music stimuli for use in future research. To this end, we calculated average ratings for each song on the following variables: naming (% correct), familiarity, valence, arousal, liking, and autobiographical salience (% of times the song evoked a memory). We also calculated Pearson’s correlations to identify relationships between these variables for each clip.

We sought to further classify and describe these stimuli by conducting a cluster analysis to identify whether the stimuli naturally clustered into certain groups based on the ratings. To this end, we conducted a k-means cluster analysis to group the clips based on the normative ratings. First, we sought to identify the optimal number of clusters based on the data. To do this, we first standardized the ratings by converting them from raw ratings to* z* scores. Then we used the silhouette method to identify the number of clusters using the “NbClust” function from the *NbClust* package in R (Charrad et al., 2014). The silhouette method measures the similarity of (or, the distance between) objects within each cluster as compared with the similarity of items between clusters (Kaufman & Rousseeuw, 2005). A higher silhouette value indicates better clustering, such that within-cluster similarity is maximized while between-cluster similarity is minimized. After determining the optimal number of clusters, we performed k-means clustering using the “kmeans” function in R.

### Results

#### Normative ratings

Normative ratings for all 141 stimuli can be found in the OSF repository. To give some illustrative examples, the clip that received the highest naming score was “Macarena” (Bayside Boys Mix) by Los del Rio (1996; 96%) and the clip with the lowest naming score was “Stranger on the Shore” by Acker Bilk (1962; 0%). The clip with the highest rated familiarity was “I Will Always Love You” by Whitney Houston (1993; *M* = 5.80) and the clip with the lowest familiarity was “Stranger on the Shore” by Acker Bilk (1962; *M* = 1.43). The clip with the highest-rated emotional valence was “Happy” by Pharrell Williams (2014; *M* = 6.26) and the clip with the lowest-rated emotional valence was “Alone Again (Naturally)” by Gilbert O’Sullivan (1972; *M* = 2.90). The clip with the highest emotional arousal was “Baby Got Back” by Sir Mix-a-Lot (1992; *M* = 6.26) and the clip with the lowest emotional arousal was “I Can’t Stop Loving You” by Ray Charles (1962; *M* = 2.06). The clip with the highest liking rating was “Billie Jean” by Michael Jackson (1983; *M* = 6.03) and the clip with the lowest liking rating was “Ballad of the Green Berets” by Barry Sadler (1966; *M* = 2.96).

**Fig. 1:**
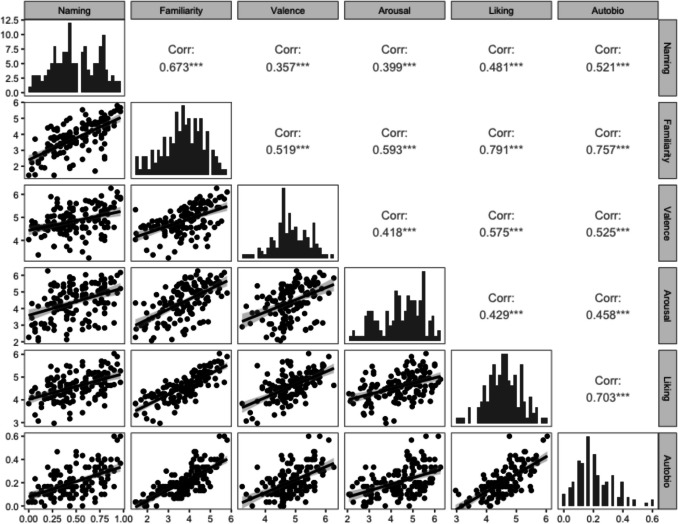
Correlations between the measured variables. Lower panels depict scatterplots with a linear regression line superimposed. Gray band surrounding regression lines indicates 95% confidence interval. Diagonals depict histograms indicating the frequency for each response variable. Upper panels show the *r* values for correlations between each variable

The clip that evoked the most autobiographical memories was “Billie Jean” by Michael Jackson (1983; 60%); there were four clips that evoked zero autobiographical memories: “Foolish Games” by Jewel (1997), “Little Things Mean a Lot” by Kitty Kallen (1954), “Tossin’ and Turnin’” by Bobby Lewis (1961), and “Wanted” by Perry Como (1954). We sought to investigate relationships among these variables by calculating Pearson’s correlations for each pair of variables (averaged for each piece of music). There were significant positive correlations between all measured variables. Some of the strongest correlations included familiarity with liking, autobiographical salience, and naming, while emotional features (arousal and valence) showed weaker correlations with the other measured variables. See Fig. 1 for a graphical depiction of these data.

#### Cluster analysis

**Fig. 2:**
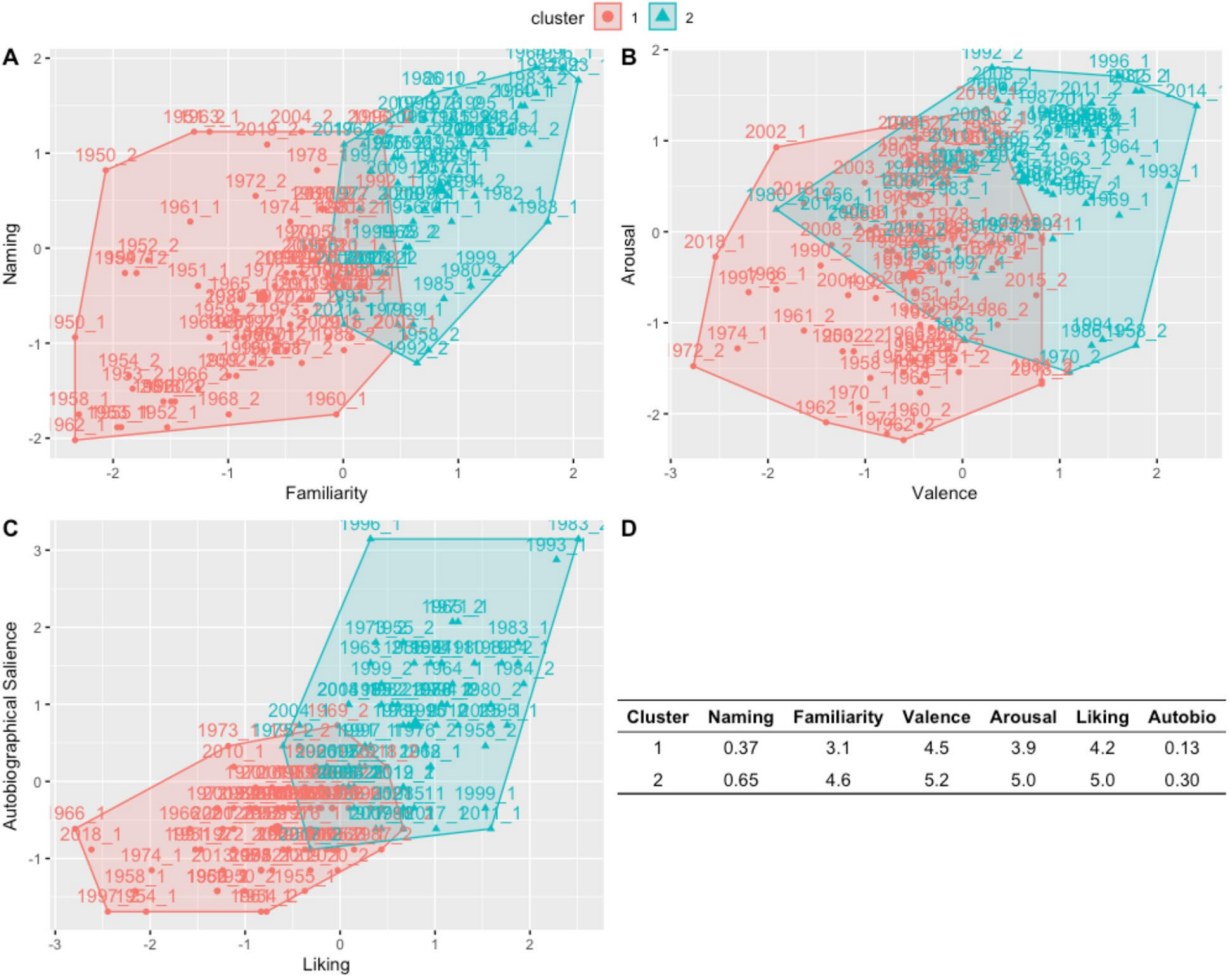
Cluster visualization. Clusters 1 and 2 are mapped on to naming and familiarity (**A**), arousal and valence (**B**), and Autobiographical salience and liking (**C**). **D** Mean values for each cluster on each variable. (Color figure online)

We first used the silhouette method to identify the optimal number of clusters based on our data, which identified *two* as the optimal number. We then performed k-means clustering with a predetermined cluster number of two. There were 77 music clips in Cluster 1 and 64 music clips in Cluster 2 (the full list of stimuli and the cluster to which each belongs can be found in the Supplemental Materials). Cluster 1 consisted of stimuli that were less familiar, had less autobiographical salience, were less frequently named, and had moderate valence, arousal, and liking. In Contrast, Cluster 2 consisted of stimuli that were highly familiar, had a higher frequency of autobiographical salience and naming, and had high valence, arousal, and liking. See Fig. 2 for a graphical depiction of these clusters and the means for each cluster on each rating scale.

These clusters are based on data for all participants, who span a wide range of ages. However, it is possible that certain stimuli may be more likely to cluster together only for participants of similar ages. To address this, we split our sample into three age bins: Younger adults (<35 years), middle-aged adults (35 to 60 years), and older adults (older than 60 years). We then reran this cluster analysis for each group to identify whether there were consistencies in the stimuli that clustered together. For each age group, we identified two clusters that directly mapped on to the clusters described above: One cluster contained unfamiliar stimuli with low autobiographical salience, and one cluster contained familiar stimuli with high autobiographical salience. We then sought to identify if there were any stimuli that were in the highly familiar cluster across all three age bins: We found 32 stimuli that were in the familiar cluster across age groups. This suggests that certain stimuli have intergenerational autobiographical salience—for example, songs like “Billie Jean” by Michael Jackson, “Macarena” by Los del Rio, “Uptown Funk” by Mark Ronson, and “Surfin’ U.S.A.” by The Beach Boys—all have high autobiographical salience across age groups. See Supplemental Materials for the list of stimuli including which cluster each stimulus falls into.

### Interim discussion

In our first study, we collected normative data on a set of popular music cues taken from the Billboard Hot 100 year-end charts from 1950–2021. Overall, around half of the musical cues fell into a cluster of stimuli that were highly recognized and had high autobiographical salience. Future research on music-evoked autobiographical memories could more specifically select stimuli from this list that are highly likely to be associated with memories; furthermore, these normative data will allow for comparisons between stimuli that are controlled for popularity at the time (e.g., two stimuli that were high on the Billboard charts) but differ in their current familiarity and recognizability. Finally, we identified a subset of stimuli that are highly familiar and highly autobiographically salient across age groups. These stimuli could be selected by researchers who want to present identical stimuli to all participants that are highly likely to evoke autobiographical memories.

## Study 2: Characterizing popular movie cues

In Study 2, we sought to also characterize a set of popular movie stimuli to use as autobiographical memory cues. We collected data from a new sample, but otherwise, the methods are identical to that of Study 1.

### Method

#### Participants

Participants (*N* = 128; 64 women, 64 men) were recruited online using Prolific, an online platform for participant recruitment (Palan & Schitter, 2018). Participation was restricted to workers who are native English speakers, live in the United States, and have completed at least 100 previous Prolific tasks and obtained approval ratings of at least 95%. Our goal was to recruit participants with evenly distributed ages between 20 and 85 years of age using a stratified sampling method. Approximately 20 participants were recruited in each of the following 10-year age bins: 20–29, 30–39, 40–49, 50–59, 60–69, and 70–85 years old.

#### Stimuli

Stimuli consisted of popular movie clips. Clips were selected to be comparable to music clips in terms of popularity. First, we identified the top 10 US box-office grossing films from 1950–2021. Given that the goal of this stimulus set is to target the “reminiscence bump” period of life, we removed any movies that were targeted directly at young children (e.g., *Frozen*). Family-oriented movies targeted at a broader audience, however, were included in the set (e.g., *Harry Potter and the Sorcerer’s Stone*). To minimize participant fatigue, we chose *two* films for each year to include in our stimulus set that would be presented to participants. Therefore, the total set consisted of 144 stimuli (two movie clips per year for 72 years). Each stimulus was clipped to a 15-s long segment that corresponded to a highly recognizable scene from the movie. Highly recognizable scenes were identified by watching the movie trailers for each film to identify prominent scenes. Again, to minimize participant fatigue, stimuli were assigned to one of four lists. Each list contained one clip from every other year, for a total of 36 clips per list. For the full list of all stimuli, including which list each clip was included in, please see the Supplemental Materials.

#### Procedure, data quantification, and analysis

Procedures, data quantification, and analysis are identical to that of Study 1. The only difference between the two studies was that they recruited different samples of participants and used different stimuli (musical clips in Study 1 and movie clips in Study 2).

### Results

#### Normative ratings

**Fig. 3:**
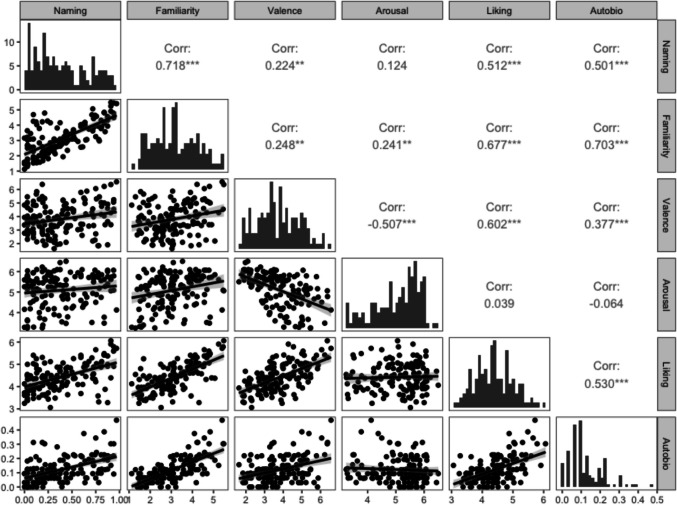
Correlations between the measured variables. Lower panels depict scatterplots with a linear regression line superimposed. Gray band surrounding regression lines indicates 95% confidence interval. Diagonals depict histograms indicating the frequency for each response variable. Upper panels show the *r* values for correlations between each variable

Normative ratings for all 144 stimuli can be found in the OSF repository. To give some illustrative examples, the clip that received the highest naming score was *Titanic* (1997; 96%) and the clips with the lowest naming scores were *Quo Vadis* (1951), *Cinerama Holiday* (1955), *The Bible: In the Beginning* (1966), and *Transformers: Dark of the Moon* (2011) (all 0% correct). The clip with the highest rated familiarity was *E.T The Extra-Terrestrial* (1982; *M* = 5.48) and the clip with the lowest familiarity was *Cinerama Holiday* (1955; *M* = 1.12). The clip with the highest-rated emotional valence was *Titanic* (1997; *M* = 6.56), and the clip with the lowest-rated emotional valence was *Fatal Attraction* (1987; *M* = 1.62). The clip with the highest emotional arousal was *I Am Legend* (2007; *M* = 6.5), and the clip with the lowest emotional arousal was *South Pacific* (1958; *M* = 3.24). The clip with the highest liking rating was *E.T. The Extra-Terrestrial* (1982; *M* = 6.06), and the clip with the lowest liking rating was *To Kill A Mockingbird* (1962; *M* = 3.06). The clip that evoked the most autobiographical memories was *Titanic* (1997; 46%); there were 10 clips that evoked zero autobiographical memories. We sought to investigate relationships among these variables by calculating Pearson’s correlations for each pair of variables (averaged for each piece of music). The relationships differed slightly from the music clips. Namely, arousal was not significantly correlated with naming, liking, or autobiographical salience; all other variables were significantly positively correlated with one another, except arousal and valence, which were negatively correlated. See Fig. 3 for a graphical depiction of these data.

#### Cluster analysis

**Fig. 4:**
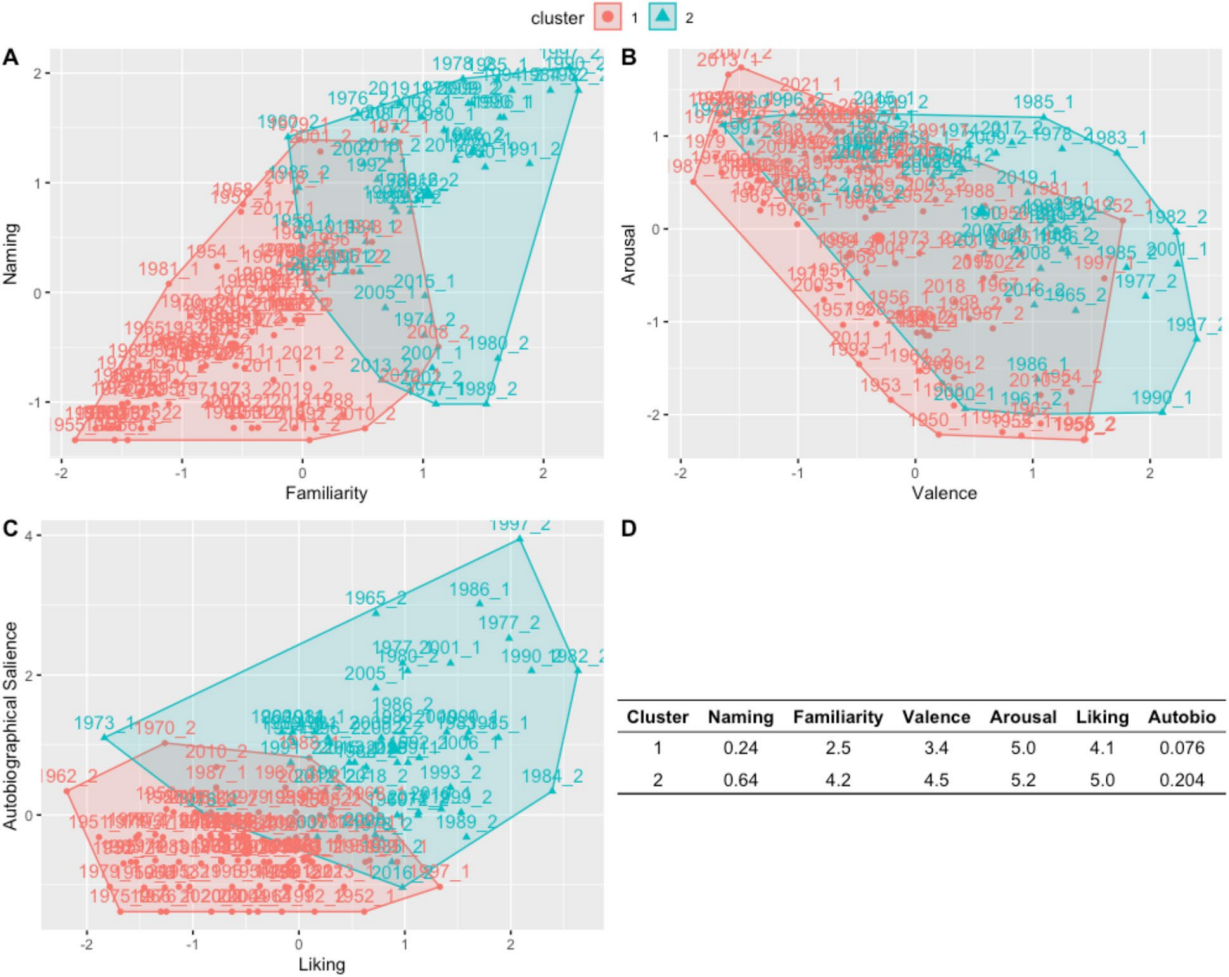
Cluster visualization. Clusters 1 and 2 are mapped on to naming and familiarity (**A**), arousal and valence (**B**), and Autobiographical salience and liking (**C**). **D** Mean values for each cluster on each variable. (Color figure online)

We first used the silhouette method to identify the optimal number of clusters based on our data, which (as with the musical stimuli) identified *two* as the optimal number. We then performed k-means clustering with a predetermined cluster number of two. There were 93 music clips in Cluster 1 and 51 music clips in Cluster 2 (the full list of stimuli and the cluster to which each belongs can be found in the Supplemental Materials). Cluster 1 consisted of stimuli that were less familiar, had less autobiographical salience, were less frequently named, and had moderate valence and liking, but high arousal. In contrast, Cluster 2 consisted of stimuli that were highly familiar, had a higher frequency of autobiographical salience and naming, and had high valence, arousal, and liking. See Fig. 4 for a graphical depiction of these clusters and the means for each cluster on each rating scale.

As with the music stimuli, we sought to identify specific stimuli that are highly familiar and autobiographically salient across all ages. To this end, we conducted the same cluster analysis on subgroups of our participants (younger adults <35 years old, middle-aged adults between 35 and 60, and older adults above 60 years of age). For each subgroup, we identified the same two types of clusters: One cluster consisted of familiar and autobiographically salient stimuli, while a second cluster consisted of unfamiliar and nonsalient stimuli. We identified 26 stimuli that fell into the “familiar” cluster for all three age groups, suggesting that these stimuli would be effective memory cues for participants across the lifespan. These included movie clips such as *The Godfather*, *The Empire Strikes Back*, *Twister*, and *Ghostbusters*. See Supplemental Materials for a full list of stimuli and their clusters.

## Interim discussion

In Study 2, we sought to characterize a set of popular movie cues for use in future research. We created a set of 144 popular movie cues from 1950–2021 and collected normative data on these stimuli. Differing from the music stimuli, for movies, the relationship between emotional arousal and the other variables was less straightforward—for movies, arousal tended to be negatively correlated with emotional valence, which contrasts with much prior research on music (which typically tends to find positive correlations between emotional arousal and emotional valence). It is possible that films are more likely to depict highly arousing, but negatively valenced scenes than music, especially popular music from the Billboard charts. Prior work has found the same for poetry and visual artwork, that these two categories of art are more likely to have the combination of high-arousal and low-valence than classical, jazz, and electronic music, as well (Mehl et al., 2023). Our data also indicated that there were two clusters of stimuli, one of which were highly familiar and the other of which were less familiar; this pattern replicated the pattern that was found for the musical cues. As with the musical cues, we also identified a subset of movie stimuli that appear to be familiar and autobiographically salient across all age groups.

## Study 3: Comparing memories evoked by music and movie cues

The goals of the prior two studies were to create and characterize two stimulus sets (music and movies) to provide as a resource for future research. While those served a methodological aim, we also sought to investigate our empirical aim, which is to identify differences between the properties of the two stimuli as memory cues. Namely, we wanted to investigate whether the content of memories evoked by music and movies differed, whether the subjective ratings of these stimuli differed, and whether the distribution of memories for stimuli across the lifespan (i.e., the shape of the reminiscence bumps) differed.

### Method

Here, we combined the datasets from Studies 1 and 2 to directly compare between ratings of popular music clips and popular movie clips. Participants, stimuli, and procedures for the two datasets are described above. The two groups did not significantly differ in terms of age, *t*(246) = 1.24, *p* = .21, or gender, χ^2^ < 0.001, *p* = 1.

#### Data quantification

In addition to the data quantification described in Studies 1 and 2, here, we sought to investigate the memory content produced in response to musical and movie clips. For stimuli that evoked autobiographical memories, participants were asked to provide brief descriptions of those memories. The memories were then assessed using the Linguistic Inquiry and Word Count software (LIWC; Pennebaker et al., 2015). The LIWC is a dictionary matching method which categorizes each word in a text and calculates a percentage of words in each category (number of words in a category divided by the total number of words; Pennebaker et al., 2015; Tausczik & Pennebaker, 2010). These categories range from parts of speech to affective dimensions of a text and counts the number of words present in a. To limit the number of statistical tests (the LIWC outputs hundreds of variables), we selected a subset of these for our analysis based on our prior work (Belfi et al., 2022; Pearson et al., 2023). Namely, we used the “higher order” categories reflecting the content of the text which included: Affective words (e.g., “happy,” “cried”), social words (e.g., “talk,” “they”), cognitive words (e.g., “cause,” “know”), and perceptual words (e.g., “see,” “listen”).

For the reminiscence bump analysis, we first calculated the “stimulus-specific age” (SSA) for each stimulus for each participant. Prior work has utilized SSA to denote “song-specific age” for investigating music-evoked autobiographical memories. Here, we modified this term to mean “stimulus-specific age” as we are investigating both movies and music. SSA is a measure of how old an individual was when each stimulus was released, and is calculated by subtracting each participant’s birth year from the year that the song was featured in the Billboard charts or the year when the movie was released (Holbrook & Schindler, 1989; Jakubowski et al., 2020). Negative SSA values denote stimuli that were released prior to a participant’s birth, whereas positive SSA values denote stimuli that were released at the participant’s specific years of age.

#### Analysis

First, we analyzed the content of the memories. We first did this by conducting a multivariate analysis of variance (MANOVA) on the LIWC data, with four dependent variables from the LIWC output (affective, social, cognitive, and perceptual words) and condition (music, movies) as the independent variable. All variables were first averaged across all memories within each participant. We averaged within participants to control for potential differences in the number of memories between groups. We used the same methodological approach on the subjective ratings of the stimuli (familiarity, valence, arousal, and liking) as well as the percentage of correct naming.

To analyze the reminiscence bump data, we first aggregated the ratings into SSA bins of 15 years (e.g., 5 to 19 years, 20 to 35 years). It is important to note that not all participants provided ratings in each bin; for example, only participants who are 65 years of age or older would provide ratings for stimuli in the SSA bin 65-85, and only younger participants would provide ratings for stimuli in the SSA bin −55 to −40 (which denotes stimuli that were popular 55 to 40 years prior to birth). To analyze the autobiographical memory data and naming data (both of which were scored in a binary 0 vs. 1 fashion) we calculated generalized linear mixed-effect models using the “glmer” function from the *lme4* package in R (Bates et al., 2015). Each model included fixed effects for condition (music vs. movies), SSA, and the interaction between condition and SSA, and random intercepts for participants and stimuli. For familiarity and liking ratings, which were not binary, we conducted standard linear mixed-effect models with the same fixed and random effects as described above. For all models, the “anova” function from the package *car* was used to conduct significance tests (Fox & Weisberg, 2011), and pairwise comparisons were calculated using the *emmeans* package (Lenth, 2020).

## Results

### Memory content

**Fig. 5:**
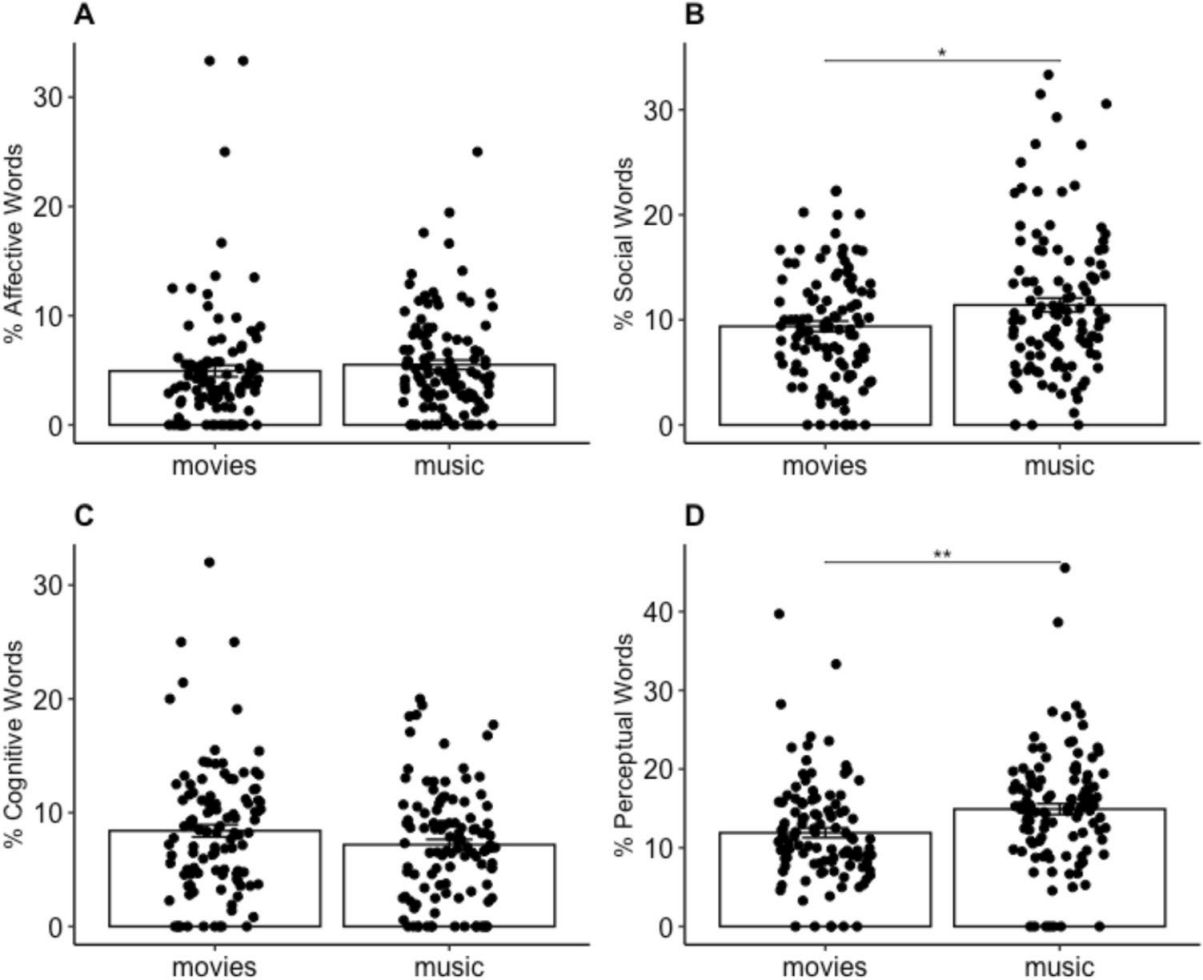
LIWC data. **A** Affective words. **B** Social words. **C** Cognitive words. **D** Perceptual words. Bar heights indicate mean, error bars indicate standard error of the mean, individual points indicate individual subjects. *Y-*axes depict the percentage of words that fall into each category (averaged across memories for each participant. *p < .05, **p < .01

We first compared whether the content of the memories evoked by music and movies differed based on output from the LIWC. We removed one participant from the dataset who was an outlier, as their reported memories were only one word long which led to the LIWC percentages being 100% for each memory. First, there was no difference in the average word counts for each group: on average, MEAMs were 17.3 words long and movie-evoked memories were 15.6 words long, *t*(221) = 1.25, *p* = .21, 95% CI [−0.93, 4.15]. The MANOVA assessing the effect of condition (music, movies) on the LIWC variables (affective, social, cognitive, and perceptual words) was significant, *F*(4,218) = 5.53, *p* < .001, η_p_^2^ = 0.09, a medium effect size. Post hoc univariate analyses of variance (ANOVAs) for each dependent variable revealed significant effects of condition on social words, *F*(1,221) = 6.03, *p* = .01, and perceptual words, *F*(1,221) = 10.65, *p* = .001. Post hoc pairwise comparisons, Bonferroni corrected for multiple comparisons, conducted following the significant univariate ANOVAs revealed that participants in the music condition produced memories that contained a significantly greater proportion of social words than participants in the movies condition, *t*(221) = −2.45, *p* = .01, 95% CI [−3.65, −0.40]. Similarly, participants in the music condition produced memories that contained significantly more perceptual words than in the movies condition, *t*(221) = −3.26, *p* = .001, 95% CI [−4.82, −1.19]. See Fig. 5 for a graphical depiction of the data.

#### Subjective ratings

**Fig. 6:**
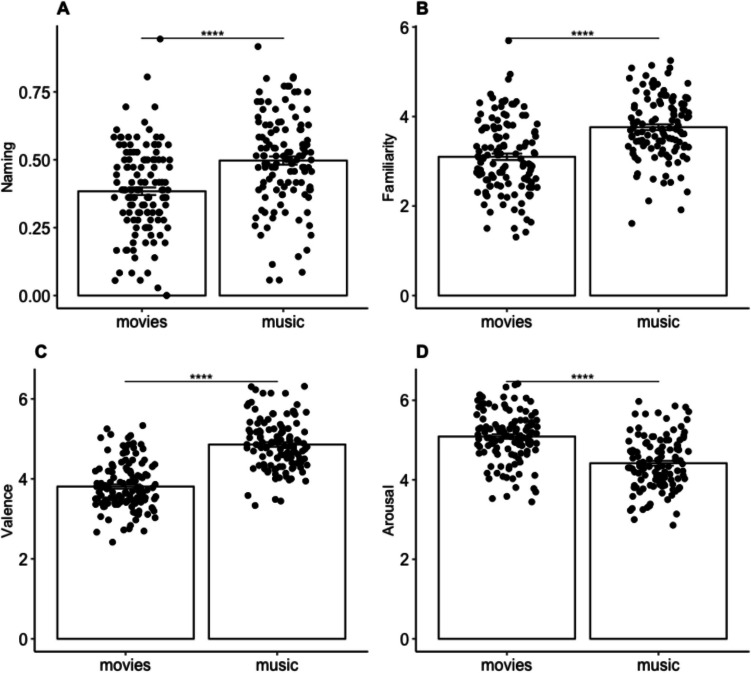
Subjective rating data. **A** Naming (proportion correct). **B** Familiarity ratings. **C** Valence ratings. **D** Perceptual ratings. Bar heights indicate mean, error bars indicate standard error of the mean, individual points indicate individual subjects. *Y*-axes depict the percentage of words that fall into each category (averaged across memories for each participant. ****p < .001

Next, we compared whether subjective ratings of the stimuli (familiarity, valence, arousal, liking) as well as naming, differed between the music and movie stimuli. The MANOVA assessing the effect of condition (music, movies) on these data was significant, *F*(5,242) = 77.2, *p* < .001, η_p_^2^ = 0.61, a very large effect size. Post hoc univariate ANOVAs for each dependent variable revealed significant effects of condition on naming, *F*(1,246) = 28.62, *p* < .001, familiarity, *F*(1,246) = 46.98, *p* < .001, valence, *F*(1,246) = 183.91, *p* < .001, and arousal, *F*(1,246) = 68.59, *p* < .001. The only variable that did not differ between conditions was liking. Post hoc pairwise comparisons, Bonferroni corrected for multiple comparisons, conducted following the significant univariate ANOVAs, revealed that the musical cues were named correctly more often than the movie cues, *t*(120) = −5.35, *p* < .001, 95% CI [−0.15, −0.07], the musical cues were rated as more familiar than the movie cues, *t*(120) = −6.85, *p* < .001, 95% CI [−0.85, −0.47], the musical cues were rated as more positively valenced, *t*(120) = −13.56, *p* < .001, 95% CI [−1.20, −0.89], and the movie cues were rated as more arousing, *t*(120) = 8.27, *p* < .001, 95% CI [0.51, 0.83]. See Fig. 6 for a graphical depiction of the data.

#### Reminiscence bumps

The model looking at the autobiographical memory ratings identified an overall main effect of condition (such that the musical stimuli evoked significantly more autobiographical memories than the movie stimuli) and a main effect of SSA (such that stimuli in SSA age bins 5–19 and 20–34 evoked significantly more autobiographical memories than stimuli outside of those age bins). However, there was also a significant interaction between SSA and condition, and therefore, given the presence of a significant interaction, we will focus on those results over the main effects: Specifically, musical stimuli were associated with autobiographical memories at a higher rate than movie stimuli at SSA bins −10 to 4 (*β* = 0.95, *SE* = 0.20, *z* = 4.64, *p* < .001), 5 to 19 (*β* = 1.15, *SE =* 0.17, *z* = 6.62, *p* < .001), and 20 to 34 (*β* = 0.91, *SE* = 0.18, *z* = 4.93, *p* < 0.001). Given that musical stimuli were overall rated as more familiar than movie stimuli (see “Subjective Rating” analysis section above), we wanted to investigate whether the autobiographical memory effect was primarily driven by differences in familiarity. To this end, we reran this model and added familiarity as a covariate. Results indicated that still, musical cues evoked a higher proportion of autobiographical memories than movie cues for SSA bins 5 to 19 (*β* = 0.52, *SE* = 0.19, *z* = 2.72, *p* = .006) and 20 to 34 (*β* = 0.62, *SE* = 0.20, *z* = 3.04, *p* = .002), even after controlling for familiarity.

**Fig. 7:**
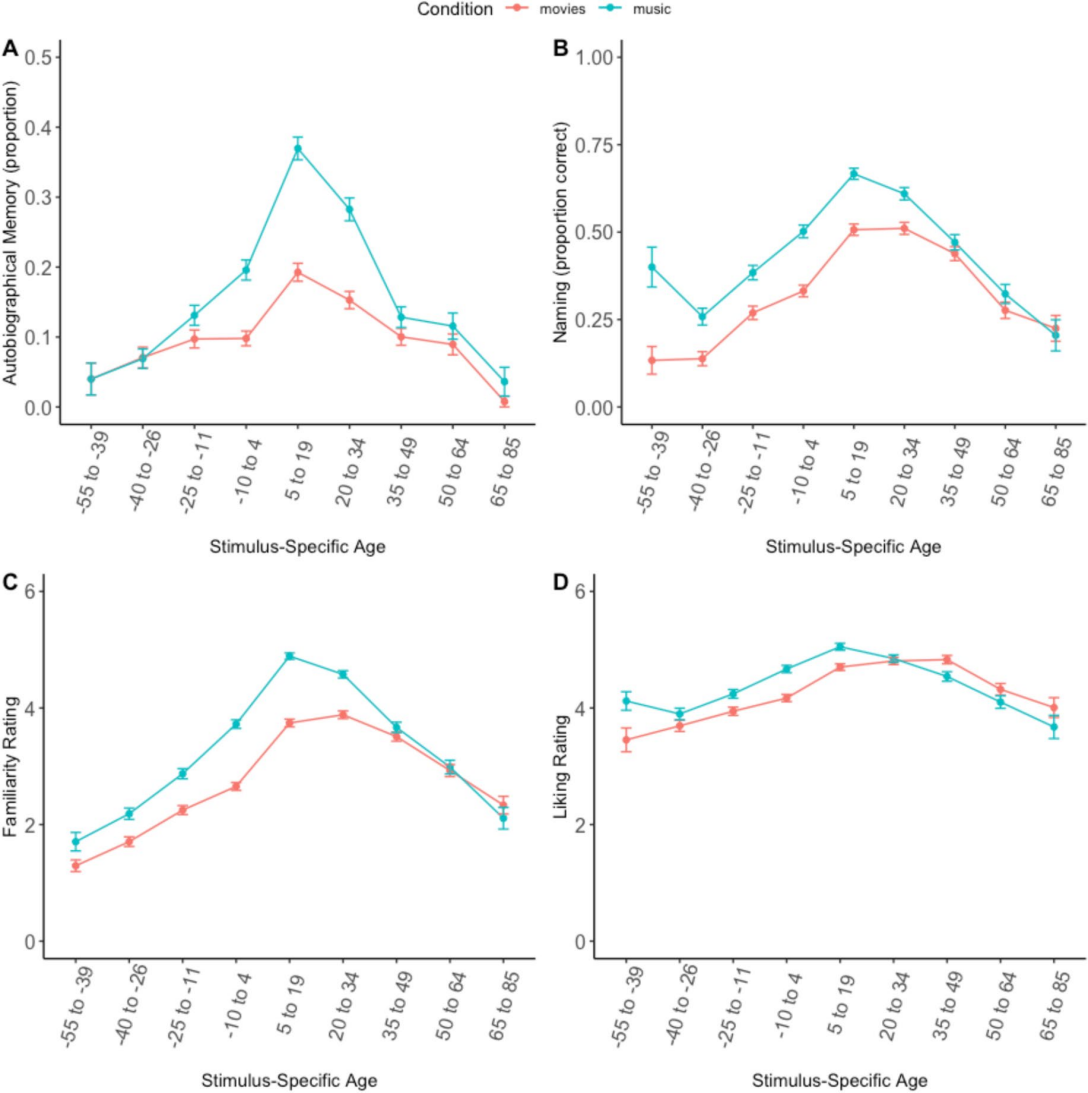
Reminiscence bump data. **A** Autobiographical memory. **B** Naming. **C** Familiarity ratings. **D** Liking ratings. Error bars indicate standard error of the mean

For naming, there were also significant main effects of condition, SSA, and a significant interaction between condition and SSA: Music stimuli were named significantly more accurately at SSA −55 to −39 (*β* = 1.82, *SE* = 0.48, *z* = 3.74, *p* < .001), −40 to −26 (*β* = 0.74, *SE* = 0.26, *z* = 2.80, *p* = .005), −25 to −11 (*β* = 0.60, *SE* = 0.19, *z* = 3.14, *p* = .005), −10 to 4 (*β* = 0.74, *SE* = 0.16, *z* = 4.53, *p* < .001), 5 to 19 (*β* = 0.80, *SE* = 0.15, *z* = 5.21, *p* < .001), and 20 to 34 (*β* = 0.48, *SE* = 0.15, *z* = 3.03, *p* = .002). However, when familiarity was added as a covariate, this effect remained significant only for SSA −55 to −39 (*β* = 1.69, *SE* = 0.52, *z* = 3.19, *p* = .001). For liking, there was no main effect of condition, a main effect of SSA, and a significant interaction between SSA and condition: musical stimuli were liked significantly more than movie stimuli at SSA −10 to 4 (*β* = 0.49, *SE* = 0.11, *z* = 4.10, *p* < .001) and 5 to 19 (*β* = 0.41, *SE* = 0.11, *z* = 3.61, *p*<0.001). For familiarity, there was a main effect of condition, a main effect of SSA, and a significant interaction between condition and SSA: musical stimuli were rated significantly more familiar than movie stimuli at SSA −25 to −11 (*β* = 0.60, *SE* = 0.13, *z* = 3.61, *p* < .001), −10 to −4 (*β* = 0.93, *SE =* 0.11, *z* = 8.00, *p* < .001), 5 to 19 (*β* = 1.04, *SE =* 0.11, *z* = 9.45, *p* < .001), and 20 to 34 (*β* = 0.62, *SE =* 0.11, *z* = 5.43, *p* < .001). See Fig. 7 for a graphical depiction of the data.

## Discussion

One of the goals of the present work was to provide normative ratings for a large set of popular film and music clips for future research on cued autobiographical memories. In doing so, we identified subsets of both movie and music stimuli that seem to be highly familiar and autobiographically salient across all age groups (e.g., “Billie Jean” by Michael Jackson as a musical example and *The Godfather* as a film example). That is, we have identified a subset of popular media stimuli that appear to be intergenerationally salient in that they evoke autobiographical memories regardless of participant age. This will provide researchers the opportunity to present participants with the exact same stimuli as autobiographical memory cues rather than selecting individual stimuli for each participant.

While *some* stimuli appear to be familiar and autobiographically salient regardless of participant age, we also identified a reminiscence bump for both movies and music. That is, media that were released around adolescence and early adulthood tended to be the most autobiographically salient. Therefore, our data provides researchers with two potential approaches for stimuli selection: On the one hand, researchers could choose stimuli that are familiar and salient across the board; on the other, selection of stimuli from the reminiscence bump period could be tailored on a participant-by-participant basis. Although we did identify a reminiscence bump for both movies and music, this bump was shown to be more pronounced for musical cues. This difference between movies and music was still significant even after controlling for familiarity—thus, the more prominent reminiscence bump for musical cues was not simply due to the musical cues being more familiar than the movies. Prior work has indicated that more familiar stimuli (including music and visual cues) are more likely to evoke autobiographical memories (Jakubowski & Francini, 2022), and therefore controlling for familiarity is an important point to consider when conducting research on music-evoked autobiographical memories. We provide both familiarity and autobiographical salience data on these stimuli, which will allow researchers to control for familiarity in their stimulus selection. However, our current evidence of a more pronounced reminiscence bump for music, despite controlling for familiarity, suggests that familiarity with a stimulus is not the sole factor influencing memory evocation.

Along with our primary goal of creating normative stimulus set of film and music cues, we also had an empirical aim to look at the possible difference between autobiographical memories evoked by movies and music. One prior study has compared memory retrieval in response to movies and music, specifically looking at reminiscence bumps (Rathbone et al., 2016). The methods of that paper were quite different from ours, as participants in the prior study were presented with a list of song/film titles and asked to choose five that were the most “personally significant.” The authors identified a reminiscence bump for personally significant songs, but not films. This mirrors our work somewhat, in that we showed a stronger reminiscence bump for music than films. However, it might be the case that presenting participants with the actual stimuli themselves, rather than titles, leads to increased memory retrieval. The prior study also reported a much lower proportion of stimuli in the reminiscence bump (around .20 vs. .40 in the present work), similarly suggesting that titles are less effective at evoking memories than sensory stimuli themselves.

In addition to looking at the reminiscence bumps, we also investigated differences between movie- and music-evoked autobiographical memories in terms of memory content and subjective ratings. Our work therefore also adds to the growing literature comparing music-evoked memories to those evoked by other stimuli. Our current findings show that MEAMs contained a significantly higher amount of social and perceptual words in comparison to movie-evoked memories. The perceptual finding replicates prior work comparing music to images of faces (Belfi et al., 2022), suggesting that music-evoked autobiographical memories contain more vivid details. Interestingly, however, prior work has indicated that face-evoked memories contain more social details than MEAMs (the reverse of the result found here). Other work found that MEAMs contain more perceptual and social words than memories evoked by television shows (Jakubowski et al., 2021). When looking at the subjective ratings of the stimuli, musical cues were found to be more positively valenced, more familiar, more recognizable (i.e., more frequently named correctly), but movie cues were rated as more emotionally arousing.

One potential theoretical explanation for why music is effective at evoking autobiographical memories is due to its emotionally evocative nature: That is, it might be that music evokes strong emotions that therefore evoke more vivid memories. For example, prior work has indicated that positive valence and high arousal is related to more vivid memory retrieval (Ford et al., 2012). Similarly, other work found that emotional strength was positively correlated with the number of autobiographical memories retrieved in response to film clips in older adults and individuals with Alzheimer’s disease (Rasmussen et al., 2021). This work suggests that emotional responses could be one mechanism through which music evokes vivid autobiographical memories. However, our data do not entirely indicate that music is more emotionally evocative than films. Here, musical cues were rated as more positively valenced and movie cues were rated as more emotionally arousing. Therefore, it is unclear that music is more emotionally potent than film clips. It is also worth noting that films are substantially longer than musical pieces and likely have more widespread fluctuations in emotional content. We do not claim that our movie clips here are representative of the emotional characteristics of the *entire* film. For example, the scene selected for the film *Titanic* depicts a positive interaction between Jack and Rose (and subsequently, the normative emotion ratings of the clip are highly positive). The film overall, however, depicts many emotionally negative scenes. There are therefore likely more “highs” and “lows” over the course of an entire film, and the normative emotion ratings cannot be used as a proxy of the emotion of the entire film (but simply the emotional content of the clip).

Another difference between music and movie stimuli is that music is often encountered incidentally—for example, while shopping in a store or visiting a bar or restaurant. In contrast, films are more likely to be encountered in a purposeful manner—for example, by going to a movie theater or watching a film at home. Therefore, the incidental exposure to music is most likely higher than films and may perhaps be one reason why music is a more effective memory cue (i.e., because it is more likely to be encountered frequently and encoded with memories in the first place). One theoretical explanation for why music may be a more effective memory cue is that it serves as a contextual cue. By being present in the background of other activities, hearing the music at retrieval may therefore trigger the original context. Related to this, individuals might be more likely to have listened to a specific song multiple times, perhaps many more times than they have seen a specific film. This idea of repeated exposure has not been tested yet in studies of music-evoked memories. By hearing the same song multiple times, sometimes across decades of one’s life, the song is potentially associated with *multiple* memories. Films, on the other hand, might be more likely to be associated with just a single, specific memory. This difference in memory specificity and multiple memories associated with a single stimulus poses an interesting potential avenue for future research.

Of course, this work is not without limitations. One limitation of the present work is that race and ethnicity information were not collected about the participants. Certain styles of music and certain films might be more popular with certain groups, and therefore, it is impossible to know how well these stimuli will generalize without having this information about our participants. To address this concern, we looked at the overall race/ethnicity distribution of participants on Prolific, where we recruited participants from the present work. We looked at the distribution for all participants who met our inclusion criteria (ages 20–85, native English speakers, live in the United States, and have completed at least 100 previous Prolific tasks and obtained approval ratings of at least 95%). Of these participants, 71% were White, 11% were Black, 5% were Asian, 7% were mixed, and 6% reported other or did not report their race. So, while we do not know the racial and ethnic information about our participants, we can infer the distribution based on the overall distribution of participants on Prolific.

An additional limitation of the present work relates to naming accuracy of the stimuli: Our musical stimuli were chosen from excerpts of the chorus or other highly recognizable parts of the songs. This was to maximize the likelihood that participants would recognize the excerpt and subsequently have memories evoked by it. In many popular songs, the lyrics of the song (particularly the lyrics of the chorus) often contain the title of the song. Therefore, our clip selection procedure resulted in most of our song clips including the title of the song—in fact, 74% of our musical clips include the song title within the lyrics. In contrast, only 9% of the movie clips said the title of the movie in the clip. Therefore, this could potentially explain (at least in part) the higher naming accuracy for music versus movie clips.

To conclude, research on music-evoked autobiographical memories is a rapidly growing area of research, with both basic and clinical research demonstrating an increasing interest in the potential for music to evoke memories. However, studies thus far have selected musical cues in an ad hoc manner, with no normative stimulus set available for researchers in this field. Here, we developed and provided normative data on a large set of musical and film cues for future work in this area, while also providing empirical evidence that music-evoked memories tend to show a more pronounced reminiscence bump and be more perceptually detailed than those evoked by films. We hope that the data and stimuli presented here provide a valuable resource for researchers studying the associations between sensory cues and autobiographical memory.

## Supplementary Information

Below is the link to the electronic supplementary material.

Supplementary file1 (DOCX 63 KB)

## Data Availability

Data and materials for all experiments are available at the following OSF repository (https://osf.io/5gtk4/?view_only=91690b3621b54f7281d11916e779ccf5) (Private link for peer review only). None of the experiments was preregistered.

## Code availability

Code is available upon request.

## Declarations

### Open practices statement

Data and materials for all experiments are available at the following OSF repository (https://osf.io/5gtk4/). None of the experiments was preregistered.

### Author note

Research reported in this publication was supported by the National Institute on Aging of the National Institutes of Health under Award Number R15AG075609. All data and stimuli reported in the current manuscript can be found in the following OSF repository (https://osf.io/5gtk4/).
