# Supplementary material for: Popular music and movies as autobiographical memory cues
Source: Mem Cognit. 2025 Aug 8;54(2):515–31. doi: 10.3758/s13421-025-01765-2 (PMC12957643; doi:10.3758/s13421-025-01765-2)
Supplement: Supplementary file 1 — Supplementary file1 (DOCX 63 KB) [file 13421_2025_1765_MOESM1_ESM.docx]

**Supplemental Materials**

Table of Contents

[Table S1 – List of Movie Stimuli 2](#_Toc203475296)

[Table S2 – List of Music Stimuli 6](#_Toc203475297)

# Table S1 – *List of Movie Stimuli*

| **List** | **Year** | **Number** | **Cluster** | **Name** |
| --- | --- | --- | --- | --- |
| 1 | 1950 | 1 | 1 | All About Eve |
| 2 | 1950 | 2 | 1 | Sampson and Delilah |
| 3 | 1951 | 1 | 2 | Quo Vadis |
| 4 | 1951 | 2 | 1 | The Great Caruso |
| 1 | 1952 | 1 | 1 | Moulin Rouge |
| 2 | 1952 | 2 | 1 | Sailor Beware |
| 3 | 1953 | 1 | 1 | How to Mary A Millionaire |
| 4 | 1953 | 2 | 1 | The Robe |
| 1 | 1954 | 1 | 1 | 20,000 Leagues Under The Sea |
| 2 | 1954 | 2 | 1 | Seven Brides for Seven Brothers |
| 3 | 1955 | 1 | 1 | Cinerama Holiday |
| 4 | 1955 | 2 | 1 | Oklahoma! |
| 1 | 1956 | 1 | 1 | Giant |
| 2 | 1956 | 2 | 1 | Trapeze |
| 3 | 1957 | 1 | 1 | Old Yeller |
| 4 | 1957 | 2 | 2 | The Bridge on the River Kwai |
| 1 | 1958 | 1 | 1 | Cat On A Hot Tin Roof |
| 2 | 1958 | 2 | 2 | South Pacific |
| 3 | 1959 | 1 | 1 | Ben Hur |
| 4 | 1959 | 2 | 1 | North by Northwest |
| 1 | 1960 | 1 | 2 | Psycho |
| 2 | 1960 | 2 | 2 | Spartacus |
| 3 | 1961 | 1 | 2 | El Cid |
| 4 | 1961 | 2 | 2 | The Parent Trap |
| 1 | 1962 | 1 | 2 | The Music Man |
| 2 | 1962 | 2 | 1 | To Kill A Mockingbird |
| 3 | 1963 | 1 | 2 | It's A Mad, Mad, Mad, Mad World |
| 4 | 1963 | 2 | 1 | The Great Escape |
| 1 | 1964 | 1 | 1 | From Russia With Love |
| 2 | 1964 | 2 | 1 | My Fair Lady |
| 3 | 1965 | 1 | 1 | Doctor Zhivago |
| 4 | 1965 | 2 | 1 | The Sound Of Music |
| 1 | 1966 | 1 | 1 | The Bible: In the Beginning |
| 2 | 1966 | 2 | 1 | Who's Afraid of Virginia Woolf |
| 3 | 1967 | 1 | 2 | Guess Who's Coming for Dinner |
| 4 | 1967 | 2 | 1 | The Dirty Dozen |
| 1 | 1968 | 1 | 1 | 2001: A Space Odyssey |
| 2 | 1968 | 2 | 2 | The Odd Couple |
| 3 | 1969 | 1 | 2 | Butch Cassidy and the Sundance Kid |
| 4 | 1969 | 2 | 2 | Midnight Cowboy |
| 1 | 1970 | 1 | 2 | M*A*S*H |
| 2 | 1970 | 2 | 1 | Patton |
| 3 | 1971 | 1 | 1 | Billy Jack |
| 4 | 1971 | 2 | 2 | The French Connection |
| 1 | 1972 | 1 | 2 | The Godfather |
| 2 | 1972 | 2 | 1 | The Poseidon Adventure |
| 3 | 1973 | 1 | 1 | The Exorcist |
| 4 | 1973 | 2 | 2 | The Sting |
| 1 | 1974 | 1 | 1 | The Towering Inferno |
| 2 | 1974 | 2 | 2 | Young Frankenstein |
| 3 | 1975 | 1 | 1 | Dog Day Afternoon |
| 4 | 1975 | 2 | 1 | Three Days of the Condor |
| 1 | 1976 | 1 | 1 | All The President's Men |
| 2 | 1976 | 2 | 2 | King Kong |
| 3 | 1977 | 1 | 2 | Star Wars |
| 4 | 1977 | 2 | 2 | Saturday Night Fever |
| 1 | 1978 | 1 | 1 | Heaven Can Wait |
| 2 | 1978 | 2 | 2 | Superman |
| 3 | 1979 | 1 | 1 | Alien |
| 4 | 1979 | 2 | 2 | Kramer vs. Kramer |
| 1 | 1980 | 1 | 2 | Airplane! |
| 2 | 1980 | 2 | 2 | The Empire Strikes Back |
| 3 | 1981 | 1 | 1 | Chariots of Fire |
| 4 | 1981 | 2 | 1 | On Golden Pond |
| 1 | 1982 | 1 | 2 | An Officer and A Gentleman |
| 2 | 1982 | 2 | 2 | E.T. The Extra-Terrestrial |
| 3 | 1983 | 1 | 2 | Flashdance |
| 4 | 1983 | 2 | 1 | Terms of Endearment |
| 1 | 1984 | 1 | 1 | Footloose |
| 2 | 1984 | 2 | 2 | Ghostbusters |
| 3 | 1985 | 1 | 1 | Back to the Future |
| 4 | 1985 | 2 | 1 | The Color Purple |
| 1 | 1986 | 1 | 1 | Ferris Bueller's Day Off |
| 2 | 1986 | 2 | 2 | Top Gun |
| 3 | 1987 | 1 | 1 | Fatal Attraction |
| 4 | 1987 | 2 | 2 | Moonstruck |
| 1 | 1988 | 1 | 2 | Crocodile Dundee II |
| 2 | 1988 | 2 | 1 | Rain Man |
| 3 | 1989 | 1 | 1 | Dead Poets Society |
| 4 | 1989 | 2 | 2 | Indiana Jones and the Last Crusade |
| 1 | 1990 | 1 | 1 | Ghost |
| 2 | 1990 | 2 | 1 | Home Alone |
| 3 | 1991 | 1 | 1 | The Naked Gun 2 1/2: The Smell of Fear |
| 4 | 1991 | 2 | 1 | Silence of the Lambs |
| 1 | 1992 | 1 | 1 | A Few Good Men |
| 2 | 1992 | 2 | 1 | Lethal Weapon 3 |
| 3 | 1993 | 1 | 1 | The Firm |
| 4 | 1993 | 2 | 1 | The Fugitive |
| 1 | 1994 | 1 | 1 | Speed |
| 2 | 1994 | 2 | 1 | The Mask |
| 3 | 1995 | 1 | 1 | Batman Forever |
| 4 | 1995 | 2 | 1 | Seven |
| 1 | 1996 | 1 | 1 | Independence Day |
| 2 | 1996 | 2 | 2 | Twister |
| 3 | 1997 | 1 | 2 | The Full Monty |
| 4 | 1997 | 2 | 1 | Titanic |
| 1 | 1998 | 1 | 2 | Deep Impact |
| 2 | 1998 | 2 | 2 | There's Something About Mary |
| 3 | 1999 | 1 | 1 | American Beauty |
| 4 | 1999 | 2 | 2 | The Matrix |
| 1 | 2000 | 1 | 1 | Cast Away |
| 2 | 2000 | 2 | 1 | Mission: Impossible 2 |
| 3 | 2001 | 1 | 1 | Harry Potter and the Sorcerer's Stone |
| 4 | 2001 | 2 | 1 | Pearl Harbor |
| 1 | 2002 | 1 | 1 | Minority Report |
| 2 | 2002 | 2 | 2 | Harry Potter and the Chamber of Secrets |
| 3 | 2003 | 1 | 2 | X2 |
| 4 | 2003 | 2 | 1 | The Last Samurai |
| 1 | 2004 | 1 | 1 | The Day After Tomorrow |
| 2 | 2004 | 2 | 1 | Troy |
| 3 | 2005 | 1 | 1 | Harry Potter and the Goblet of Fire |
| 4 | 2005 | 2 | 1 | War of the Worlds |
| 1 | 2006 | 1 | 2 | Night At The Museum |
| 2 | 2006 | 2 | 2 | Da Vinci Code |
| 3 | 2007 | 1 | 1 | Transformers |
| 4 | 2007 | 2 | 1 | I am Legend |
| 1 | 2008 | 1 | 2 | Iron Man |
| 2 | 2008 | 2 | 1 | The Dark Knight |
| 3 | 2009 | 1 | 1 | 2012 |
| 4 | 2009 | 2 | 1 | Avatar |
| 1 | 2010 | 1 | 1 | Inception |
| 2 | 2010 | 2 | 1 | The Twilight Saga: Eclipse |
| 3 | 2011 | 1 | 1 | The Hangover Part 2 |
| 4 | 2011 | 2 | 1 | Transformers: Dark of the Moon |
| 1 | 2012 | 1 | 2 | The Amazing Spider Man |
| 2 | 2012 | 2 | 2 | The Hunger Games |
| 3 | 2013 | 1 | 1 | Gravity |
| 4 | 2013 | 2 | 1 | Man of Steel |
| 1 | 2014 | 1 | 1 | Transformers: Age of Extinction |
| 2 | 2014 | 2 | 1 | X-Men: Days of Future Past |
| 3 | 2015 | 1 | 2 | Jurassic World |
| 4 | 2015 | 2 | 2 | The Martian |
| 1 | 2016 | 1 | 1 | Batman v Superman: Dawn of Justice |
| 2 | 2016 | 2 | 1 | Deadpool |
| 3 | 2017 | 1 | 2 | Star Wars: The Last Jedi |
| 4 | 2017 | 2 | 1 | Wonder Woman |
| 1 | 2018 | 1 | 1 | Aquaman |
| 2 | 2018 | 2 | 1 | Black Panther |
| 3 | 2019 | 1 | 2 | Joker |
| 4 | 2019 | 2 | 1 | Star Wars: The Rise of Skywalker |
| 1 | 2020 | 1 | 1 | Sonic the Hedgehog |
| 2 | 2020 | 2 | 1 | Tenet |
| 3 | 2021 | 1 | 2 | Godzilla vs. Kong |
| 4 | 2021 | 2 | 2 | No Time to Die |

# Table S2 – *List of Music Stimuli*

| **List** | **Year** | **Number** | **Cluster** | **Name** | **Artist** |
| --- | --- | --- | --- | --- | --- |
| 1 | 1950 | 1 | 1 | Hoop-Dee-Doo | Perry Como & The Fontane Sisters |
| 2 | 1950 | 2 | 1 | I Wanna be Loved | The Andrews Sisters |
| 3 | 1951 | 1 | 1 | Too Young | Nat King Cole |
| 4 | 1951 | 2 | 1 | Because of You | Tony Bennett |
| 1 | 1952 | 1 | 1 | Blue Tango | Leroy Anderson |
| 2 | 1952 | 2 | 1 | Wheel of fortune | The Hollywood Flames |
| 3 | 1953 | 1 | 1 | The Song from Moulin Rouge | Percy Faith |
| 4 | 1953 | 2 | 1 | Vaya con Dios | Les Paul & Mary Ford |
| 1 | 1954 | 1 | 1 | Little Things Mean a Lot | Kitty Kallen |
| 2 | 1954 | 2 | 1 | Wanted | Perry Como |
| 3 | 1955 | 1 | 1 | Cheery Pink And Apple Blossom White | Pérez Prado |
| 4 | 1955 | 2 | 2 | Rock Around the Clock | Bill Haley & His Comets |
| 1 | 1956 | 1 | 2 | Heartbreak Hotel | Elvis Presley |
| 2 | 1956 | 2 | 2 | Don't be Cruel | Elvis Presley |
| 3 | 1957 | 1 | 2 | All Shook Up | Elvis Presley |
| 4 | 1957 | 2 | 1 | Love Letters in the Sand | Pat Boone |
| 1 | 1958 | 1 | 1 | Volare (Nel blue Dipinto di blu) | Domenico Modugno & Johnny Dorelli |
| 2 | 1958 | 2 | 2 | All I Have to Do is Dream / Claudette | The Everly Brothers |
| 3 | 1959 | 1 | 1 | The Battle of New Orleans | Johnny Horton |
| 4 | 1959 | 2 | 1 | Mack the Knife | Bobby Darin |
| 1 | 1960 | 1 | 1 | Theme from A Summer Place | Percy Faith |
| 2 | 1960 | 2 | 1 | He'll Have a go | Jim Reeves |
| 3 | 1961 | 1 | 1 | Tossin' and Turnin | Bobby Lewis |
| 4 | 1961 | 2 | 1 | I Fall to Pieces | Patsy Cline & The Jordanaires |
| 1 | 1962 | 1 | 1 | Stranger on the Shore | Acker Bilk & The Leon Young String Chorale |
| 2 | 1962 | 2 | 1 | I Can't Stop Loving You | Ray Charles |
| 4 | 1963 | 1 | 1 | Sugar Shack | Jimmy Gilmer & The Fireballs |
| 3 | 1963 | 2 | 2 | Surfin' U.S.A. | The Beach Boys |
| 1 | 1964 | 1 | 2 | I Want to Hold Your Hand | The Beatles |
| 2 | 1964 | 2 | 2 | She Loves You | The Beatles |
| 3 | 1965 | 1 | 1 | Wooly Bully | Sam the Sham & The Pharaohs |
| 4 | 1965 | 2 | 2 | I Can't Help Myself (Sugar Pie Honey Bunch) | Four Tops |
| 1 | 1966 | 1 | 1 | Ballad of the green berets | Barry Sadler & Robin Moore |
| 2 | 1966 | 2 | 1 | Cherish (The assosiation Song | The Association |
| 3 | 1967 | 1 | 1 | The Sir With Love | Lulu |
| 4 | 1967 | 2 | 1 | The Letter | The Box Tops |
| 1 | 1968 | 1 | 2 | Hey Jude | The Beatles |
| 2 | 1968 | 2 | 1 | Love is Blue | Paul Mauriat |
| 3 | 1969 | 1 | 2 | Sugar, Sugar | The Archies |
| 4 | 1969 | 2 | 1 | Aquarius/Let the Sunshine In | The 5th Dimension |
| 1 | 1970 | 1 | 1 | Bridge Over Troubled Water | Simon & Garfunkel |
| 2 | 1970 | 2 | 2 | (They Long to Be) Close to You | Carpenters |
| 3 | 1971 | 1 | 2 | Joy to the World | Three Dog Night |
| 4 | 1971 | 2 | 1 | Maggie May / Reason to Believe | Rod Stewart |
| 1 | 1972 | 1 | 1 | The First Time Ever I Saw Your Face | Roberta Flack |
| 2 | 1972 | 2 | 1 | Alone Again (Naturally) | Gilbert O'Sullivan |
| 3 | 1973 | 1 | 1 | Tie a Tellow Ribbon Round the Ole Oak Tree | Tony Orlando & Dawn |
| 4 | 1973 | 2 | 2 | Bad, Bad Leroy Brown | Jim Croce |
| 1 | 1974 | 1 | 1 | The Way We Were | Barbra Streisand |
| 2 | 1974 | 2 | 1 | Seasons in the Sun | Terry Jacks |
| 3 | 1975 | 1 | 2 | Love Will Keep Us Together | Captain & Tennille |
| 4 | 1975 | 2 | 2 | Rhinestone Cowboy | Glen Campbell |
| 1 | 1976 | 1 | 1 | Silly Love Songs | Paul McCartney & Wings |
| 2 | 1976 | 2 | 2 | Don't Go Breaking My Heart | Elton John & Kiki Dee |
| 3 | 1977 | 1 | 2 | Tonight's the Night (Gonna be Alright) | Rod Stewart |
| 4 | 1977 | 2 | 1 | I Just Want to Be Your Everything | Andy Gibb |
| 1 | 1978 | 1 | 1 | Shadow Dancing | Andy Gibb |
| 2 | 1978 | 2 | 2 | Night Fever | Bee Gees |
| 3 | 1979 | 1 | 2 | My Sharona | The Knack |
| 4 | 1979 | 2 | 1 | Bad Girls | Donna Summer |
| 1 | 1980 | 1 | 2 | Call Me | Blondie |
| 2 | 1980 | 2 | 2 | Another Brick in the Wall, Part II | Pink Floyd |
| 4 | 1981 | 2 | 1 | Endless Love | Diana Ross & Lionel Richie |
| 1 | 1982 | 1 | 2 | Physical | Olivia Newton-John |
| 2 | 1982 | 2 | 2 | Eye of the Tiger | Survivor |
| 3 | 1983 | 1 | 2 | Every Breath You Take | The Police |
| 4 | 1983 | 2 | 2 | Billie Jean | Michael Jackson |
| 1 | 1984 | 1 | 2 | When Doves Cry | Prince |
| 2 | 1984 | 2 | 2 | What's Love Got to Do with It | Tina Turner |
| 3 | 1985 | 1 | 2 | Carless Whisper | George Michael |
| 4 | 1985 | 2 | 2 | Like a Virgin | Madonna |
| 1 | 1986 | 1 | 2 | That's What Friends Are For | Dionne Warwick |
| 2 | 1986 | 2 | 1 | Say You, Say Me | Lionel Richie |
| 3 | 1987 | 1 | 2 | Walk Like An Egyptian | The Bangles |
| 4 | 1987 | 2 | 1 | Alone | Heart |
| 1 | 1988 | 1 | 2 | Faith | George Michael |
| 2 | 1988 | 2 | 1 | Need You Tonight | INXS |
| 3 | 1989 | 1 | 1 | Look Away | Chicago |
| 4 | 1989 | 2 | 1 | My Perogative | Bobby Brown |
| 1 | 1990 | 1 | 2 | Hold On | Wilson Phillips |
| 2 | 1990 | 2 | 1 | It Must Have Been Love | Roxette |
| 3 | 1991 | 1 | 2 | (Everything I Do) I Do It for You | Brandy |
| 4 | 1991 | 2 | 1 | I Wanna Sex You Up | Color Me Badd |
| 1 | 1992 | 1 | 1 | End of the Road | Boyz II Men |
| 2 | 1992 | 2 | 2 | Baby Got Back | Sir Mix-a-lot |
| 3 | 1993 | 1 | 2 | I Will Always Love You | Whitney Houston |
| 1 | 1994 | 1 | 2 | The Sign | Ace of Base |
| 2 | 1994 | 2 | 2 | I Swear | All-4-One |
| 3 | 1995 | 1 | 2 | Gangsta's Paradise | Coolio & Kylian Mash |
| 4 | 1995 | 2 | 2 | Waterfalls | TLC |
| 1 | 1996 | 1 | 2 | Macarena | Los del Rio |
| 2 | 1996 | 2 | 1 | One Sweet Day | Mariah Carey & Boyz II Men |
| 3 | 1997 | 1 | 2 | Candle in the Wind 1997 / Something About the Way You Look Tonight | Elton John |
| 4 | 1997 | 2 | 1 | You Were Meant for Me / Foolish Games | Jewel |
| 1 | 1998 | 1 | 1 | Too Close | Next |
| 2 | 1998 | 2 | 1 | The Boy Is Mine | Brandy & Monica |
| 3 | 1999 | 1 | 2 | Believe | Cher |
| 4 | 1999 | 2 | 2 | No Scrubs | TLC |
| 1 | 2000 | 1 | 1 | Breathe | Faith Hill |
| 3 | 2001 | 1 | 1 | Hanging by a Moment | Lifehouse |
| 4 | 2001 | 2 | 1 | Fallin | Alicia Keys |
| 1 | 2002 | 1 | 1 | How You Remind Me | Nickelback |
| 2 | 2002 | 2 | 1 | Foolish | Ashanti |
| 3 | 2003 | 1 | 1 | In da Club | 50 Cent |
| 4 | 2003 | 2 | 1 | Ignition | R. Kelly |
| 1 | 2004 | 1 | 2 | Yeah! | Usher |
| 2 | 2004 | 2 | 1 | Burn | Usher |
| 3 | 2005 | 1 | 1 | We Belong Together | Mariah Carey |
| 4 | 2005 | 2 | 1 | Hollaback Girl | Gwen Stefani |
| 1 | 2006 | 1 | 2 | Bad Day | Daniel Powter |
| 2 | 2006 | 2 | 2 | Temperature | Sean Paul |
| 3 | 2007 | 1 | 2 | Irreplaceable | Beyoncé |
| 4 | 2007 | 2 | 2 | Umbrella | Rhianna |
| 1 | 2008 | 1 | 2 | Low | Flo Rida |
| 2 | 2008 | 2 | 1 | Bleeding Love | Leona Lewis |
| 3 | 2009 | 1 | 1 | Boom Boom Pow | Black Eyed Peas |
| 4 | 2009 | 2 | 2 | Poker Face | Lady Gaga |
| 1 | 2010 | 1 | 1 | Tik Tok | Kesha |
| 2 | 2010 | 2 | 2 | Need You Now | Lady A |
| 3 | 2011 | 1 | 2 | Rolling in the Deep | Adele |
| 4 | 2011 | 2 | 2 | Party Rock Anthem | LMFAO |
| 1 | 2012 | 1 | 2 | Somebody That I Used to Know | Gotye |
| 2 | 2012 | 2 | 2 | Call Me Maybe | Carly Rae Jepsen |
| 3 | 2013 | 1 | 2 | Thrift Shop | Macklemore & Ryan Lewis |
| 4 | 2013 | 2 | 1 | Blurred Lines | Robin Thicke |
| 1 | 2014 | 1 | 2 | Happy | Pharrell Williams |
| 2 | 2014 | 2 | 1 | Dark Horse | Katy Perry ft. Juicy J |
| 3 | 2015 | 1 | 2 | Uptown Funk | Mark Ronson ft. Bruno Mars |
| 4 | 2015 | 2 | 1 | Thnking Out Loud | Ed Sheeran |
| 1 | 2016 | 1 | 1 | Love Yourself | Justin Bieber |
| 2 | 2016 | 2 | 1 | Sorry | Justin Bieber |
| 3 | 2017 | 1 | 2 | Shape of You | Ed Sheeran |
| 4 | 2017 | 2 | 2 | Despacito | Luis Fonsi ft. Daddy Yankee |
| 1 | 2018 | 1 | 1 | God's Plan | Drake |
| 2 | 2018 | 2 | 1 | Perfect | Ed Sheeran |
| 3 | 2019 | 1 | 2 | Old Town Road | Lil Nas X ft. Billy Ray Cyrus |
| 4 | 2019 | 2 | 1 | Sunflower | Post Malone & Swae Lee |
| 1 | 2020 | 1 | 1 | Blidning Lights | The Weeknd |
| 2 | 2020 | 2 | 1 | Circles | Post Malone |
| 3 | 2021 | 1 | 2 | Levitating | Dua Lipa |
| 4 | 2021 | 2 | 1 | Save Your Tears | The Weeknd |
